# Peripheral IFN-ɣ Production after Blood Stimulation with Different Mycobacterial Antigens in Goats Vaccinated against Paratuberculosis

**DOI:** 10.3390/vaccines10101709

**Published:** 2022-10-13

**Authors:** Miguel Fernández, Marcos Royo, Noive Arteche-Villasol, M. Carmen Ferreras, Julio Benavides, Valentín Pérez

**Affiliations:** 1 Departamento de Sanidad Animal, Facultad de Veterinaria, Campus de Vegazana, Universidad de León, 24071 León, Spain; 2 Departamento de Sanidad Animal, Instituto de Ganadería de Montaña (CSIC-ULE), Finca Marzanas, 24346 Grulleros, León, Spain

**Keywords:** paratuberculosis, vaccination, ruminants, *Mycobacterium avium* subspecies *paratuberculosis*, interferon-gamma, immune response, tuberculosis, antigens

## Abstract

Vaccination can be an efficient method for the control of paratuberculosis in ruminants. However, the official tuberculosis control tests cross-interfere with the animals vaccinated against paratuberculosis. In order to test and compare new antigens that could solve this problem, the production of interferon-gamma (IFN-γ) in peripheral blood at different post-vaccination days in experimental kids and adult goats, in field conditions, using the avian and bovine purified protein derivative (PPD), the johnin, two peptide cocktails of *Mycobacterium bovis* (PC-EC and PC-HP) and the antigens VK 055 and VK 067 of *Mycobacterium avium* subspecies *paratuberculosis* (Map) has been analyzed in vitro. The non-specific production of IFN-γ was observed after blood stimulation with the PC-EC and PC-HP cocktail in any sample from vaccinated animals, whereas it was detected when bovine PPD was used. These results support the possible use of these new *Mycobacterium bovis* antigens in the in the differentiation of animals vaccinated against paratuberculosis or infected with tuberculosis by improving the specificity of bovine PPD. In contrast, the two Map antigens tested in this study did not improve the sensitivity of johnin or avian PPD in the detection of vaccinated or Map-infected goats.

## 1. Introduction

Paratuberculosis, caused by *Mycobacterium avium* subspecies *paratuberculosis* (Map), is a chronic infectious disease of ruminants characterized by a granulomatous enteritis with progressive loss of weight and diarrhea. It is distributed worldwide and has a great impact on livestock production [1]. Along the years, several strategies have been proposed for controlling this infection, with variable success, and, among them, vaccination has been considered an effective method in terms of benefits and costs [2]. However, in many countries, its use is prohibited due to the interference with the immune-based diagnostic tests employed in the campaigns for tuberculosis eradication [2,3]. These programs, which in Spain are annually carried out in cattle and goats, use the single intradermal tuberculin test (IDTB) as the main diagnostic technique for tuberculosis, complemented with the interferon-gamma release assay (IGRA) [4]. Although cross-reactions tend to diminish with time, their existence among the animals vaccinated against paratuberculosis has been observed with both tests [2,5,6,7,8]. 

The main suspected reason is the low specificity of bovine purified protein derivative (PPD), used as an antigen in these tests, obtained from the heat-treated culture of *Mycobacterium bovis* (*M. bovis*) [9]. These mycobacteria have phylogenetic similarities with Map, which makes them share common antigens [10,11].

In recent years, new antigens from *M. bovis*, potentially substitutes for classic tuberculin, have been produced and evaluated in order to improve the specificity for tuberculosis diagnosis and allow differentiating animals vaccinated or infected with paratuberculosis from those animals infected with tuberculosis, among which recombinant proteins from *M. bovis* such as ESAT-6, CFP-10, and RV3165c stand out. 

These studies have been performed in some experimental kids or calves infected with *M. bovis* [6,8,12,13,14] and Map [15], but they have been barely assessed in relation to paratuberculosis vaccination, only being investigated in a limited number of goats or cattle [6,13,14,15,16,17] under experimental conditions rather than in field or experimental studies with Map-infected or vaccinated animals for longer time and at different age range.

Therefore, this study aims to evaluate, in vitro, the efficacy of different antigens derived from Map or *M. bovis*, in comparison with johnin, bovine, and avian PPD used in the IGRA test, a technique employed in tuberculosis eradication campaigns, both in goats from a commercial farm vaccinated against paratuberculosis at different ages and in experimental kids vaccinated and experimentally infected with Map.

## 2. Materials and Methods

*Ethical Approval*

The experimental procedures with animals that constitute this study received the authorization of the experimentation animal committee from the University of León with authorization reference OEBA-ULE-016-2017 and a positive evaluation of the ethics committee CEE-IGM2017-001.

### 2.1. Animals and Experimental Design

**Figure 1:**
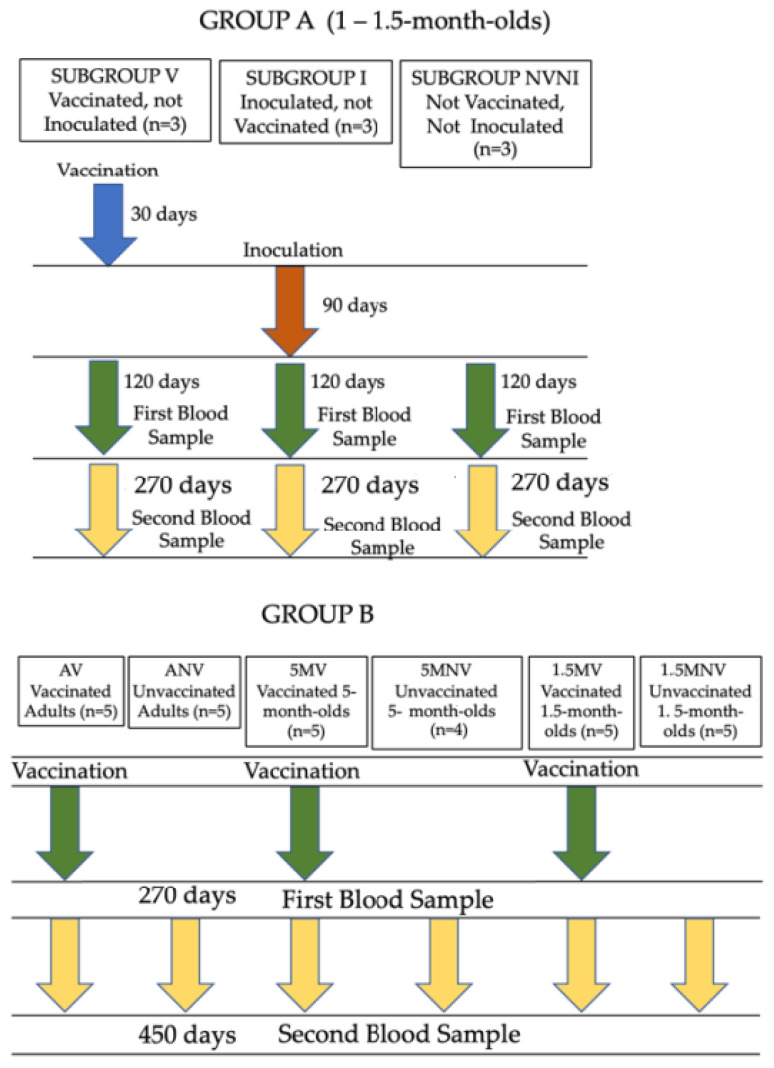
Experimental groups and animal sampling time-point.

Two experimental groups were used in this study (Figure 1). Group A consisted of nine 1- to 1.5-month-old Murciano-Granadina kids managed under experimental conditions and vaccinated with the inactivated vaccine against paratuberculosis (Gudair^®^, Vetia Animal Health, S.A., Madrid, Spain). The animals were orally challenged with a total dosage of 1.2 × 10^10^ mycobacteria, given in four doses, of the type C K-10 strain of Map, 30 days after vaccination. Three different subgroups were established within this group: vaccinated animals (V), formed by three kids that had been vaccinated, but not challenged; infected (I), composed of three kids that had been challenged with Map, but not vaccinated; and the not vaccinated not infected (NVNI) group, formed of three kids that had not undergone any treatment. In these animals, the peripheral production of IFN-γ was evaluated, using the IGRA technique, after stimulation of the blood samples with different mycobacterial antigens (detailed in Section 2.2) at 120 days post-vaccination (dpv) (90 days post-infection (dpi) and at 270 dpv (240 dpi). 

Group B consisted of Murciano-Granadina goats from a commercial dairy farm, with a free status of tuberculosis and non-clinical losses of paratuberculosis in the five past years. The animals had been vaccinated at different ages (1.5 months, 5 months, and adult animals older than 1.5 years), and six subgroups were established: vaccinated adults (AV) and unvaccinated adults (ANV), each one consisting of five vaccinated or unvaccinated adult goats, respectively; 5-month-old goat kids vaccinated (5 MV) and unvaccinated (5 MNV), composed of five goats and four goats, respectively; and finally, 1.5-month-old vaccinated (1.5 MV) or unvaccinated (1.5 MNV) kids, with each group consisting of five animals. In all cases, the animals were immunized with the same vaccine as the animals from Group A. The IFN-γ production was also assessed after stimulation, at 270 dpv and 450 dpv, of the blood samples with the antigens that will be detailed in Section 2.2.

### 2.2. Antigens

Different groups of antigens obtained from Map, *M. bovis*, and Maa were employed:

(a) purified protein derivatives obtained from heat-treated Maa or *M. bovis* cultures, known as avian and bovine PPD, respectively (CZ Veterinaria, Porriño, Pontevedra).
(b) Johnin (Neiker-Tecnalia, Derio, Vizcaya, Spain): purified protein derivative obtained from a heat-treated Map culture.
(c) PC-EC and PC-HP peptide cocktails (Thermo Fisher Scientific, Waltham, MA, USA), which are combinations of different proteins of *M. bovis*: the PC-EC cocktail has two proteins in its formulation: ESAT-6 and CFP-10, while the PC-HP has these same proteins, plus Rv-3615c. These peptide cocktails have previously been used in the diagnosis of bovine and caprine tuberculosis with satisfactory results [6,8,18].
(d) VK 055 and VK 067 antigens (Vacunek^®^, Derio, Vizcaya, Spain) purified from Map. The latter, due to availability, were used only in samples from Group A, at 4 months post vaccination (mpv) (120 dpv or 90 dpi).

### 2.3. Determination of IFN-γ Production in Peripheral Blood

The blood samples were collected in 10 mL Vacutainer^®^ tubes (Becton Dickinson, Plymouth, UK) with heparin, by jugular vein puncture. Within 4 h after collection, three 1.5 mL aliquots of blood were taken and stimulated respectively with the following compounds and concentrations: 100 µL of phosphate-buffered saline (PBS), 100 µL of bovine PPD (20 µg/mL), and 100 µL of avian PPD (20 µg/mL). In addition, another three aliquots of 0.5 mL of blood were stimulated with: 50 µL of PBS, 50 µL of the peptide cocktail PC-EC (5 µg/mL), and 50 µL of the PC-HP peptide cocktail (5 µg/mL). Finally, four aliquots of 0.5 mL of blood were stimulated with 35 µL of PBS, 35 µL of johnin (55 µg/mL), 35 µL of VK 055 (22 µg/mL), and 35 µL of VK 067 (33 µg/mL), respectively. The latter, as mentioned, only involved animals from Group A at 4 mpv.

The determination of the amounts of blood and antigens was made according to the availability of the reagents and the manufacturer’s indications. In all cases, both the peptide cocktails and the PPDs were reconstituted according to the instructions provided. All stimulations were carried out in 2 mL microcentrifuge tubes (Eppendorf, Hamburg, Germany).

The samples with the different antigens were incubated in an oven at 37 °C in a humidified atmosphere for 20 h. Once the incubation was finished, they were centrifuged at 6000 rpm for 5 min for plasma collection and stored at −20 °C for 24 h until later use.

**Figure 2:**
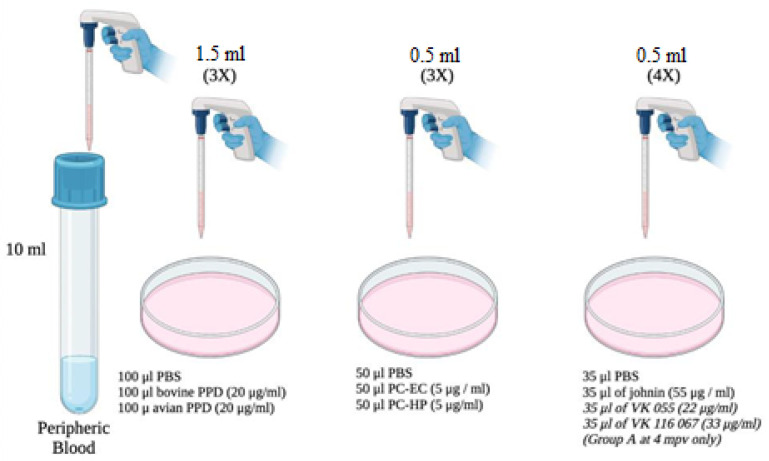
Sample preparation for the determination of IFN-γ production in peripheral blood.

The IGRA technique was carried out and duplicated on plasma, as described elsewhere [19]. When the incubations and necessary treatments had been completed, the amount of IFN-γ was assessed by reading the plates in a spectrophotometer (ELx800 Universal Microplate Reader^®^, Agilent Technologies, Santa Clara, CA, USA), at 450 nm, within 20 min after adding the braking solution. For each sample, an IFN-γ index was obtained, using the following formula: mean OD of each sample stimulated with antigen/mean OD of each sample stimulated with PBS (Figure 2).

### 2.4. Statistical Analysis

The IFN-γ production data were analyzed by means of an analysis of variance (one-way ANOVA) procedure, using GraphPad Prism 5 software (Dotmatics, San Diego, CA, USA). Normality was confirmed by Kolmogorov–Smirnov. For the establishment of differences, Tukey test analysis was performed considering a 0.05 significance level.

## 3. Results

**Figure 3:**
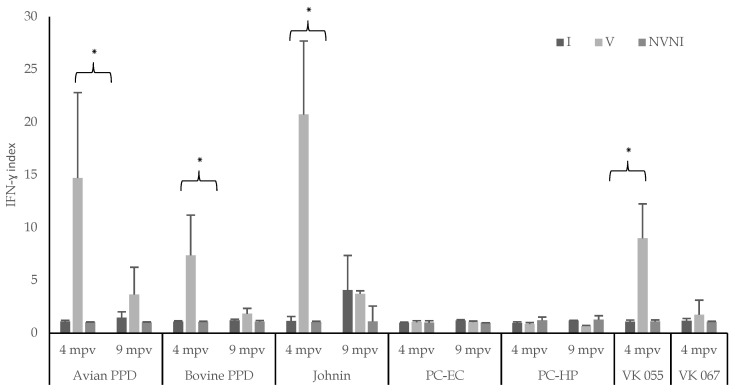
Group A. Mean and SD values of the IFN-γ index in the animals from the different subgroups, at 4 and 9 mpv, according to the antigen used for blood stimulation (avian PPD, bovine PPD, johnin, PC-EC, PC-HP, VK 055, and VK 067). mpv: months post vaccination. V: vaccinated; I: infected; NVNI: not vaccinated not infected. Error bar: standard deviation (SD). Significant difference (*).

The IFN-γ production levels at 4 and 9 mpv in the infected, vaccinated, and NVNI animals from Group A, after the stimulation of the blood samples with Maa and Map antigens, are shown in Figure 3. 

At 4 mpv, the vaccinated animals (V) presented higher mean levels (*p* < 0.05) of the IFN-γ index in the blood stimulated with avian PPD, johnin, and the VK 055 antigen, than the infected (I) and control (NVNI) subgroups, with no differences between the latter two. There were also no significant differences between the three subgroups when the blood was stimulated with the VK 067 antigen. Although IFN-γ production reached the highest levels when the blood was stimulated with johnin, this difference was not significant with the value obtained after the stimulation of blood with avian PPD, but they were with VK 055 antigen (*p* < 0.05). However, no significant differences were seen between the IFN-γ values obtained after the blood stimulation with avian PPD and with VK 055 (Figure 3).

At 9 mpv, there were also no significant differences among the subgroups for any of the antigens used (avian PPD and johnin). In the case of the infected and unvaccinated animals (I), a greater production of IFN-γ was observed when the blood was stimulated with johnin compared to avian PPD, but these differences were not significant; the difference was also greater, but not significant, when comparing the infected (I) and control (NVNI) subgroups for johnin (Figure 3).

After stimulating the blood with antigens derived from *M. bovis* (PC-EC, PC-HP, and PPD), at 4 mpv, the production of IFN-γ was only significantly higher in the vaccinated animals when the blood was stimulated with bovine PPD (*p* < 0.05). However, at 9 mpv, as with avian PPD, differences between the subgroups were no longer observed.

It is noteworthy that, unlike bovine PPD, no significant response was found in the vaccinated subgroup when the blood was stimulated with the peptide cocktails PC-EC and PC-HP in either of the two samplings performed. When comparing the IFN-γ production of the vaccinated subgroup (V) after blood stimulation with all antigens at 4 mpv, the highest values were reached after stimulation with avian PPD and johnin. These values were significantly higher than those observed after incubation with bovine PPD. At 9 mpv, only significant differences (*p* < 0.05) were observed between johnin and bovine PPD, PC-EC, or PC-HP in the subgroup of vaccinated kids (V) (Figure 3).

**Figure 4:**
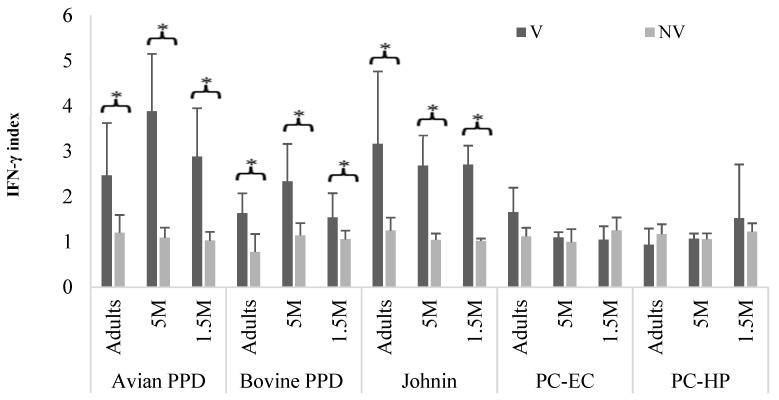
Group B. Mean and SD values of the IFN-γ index in animals of the different age subgroups, according to the antigen used to stimulate the blood, at 9 mpv. M: months old; mpv: months post vaccination; V: vaccinated; NV: not vaccinated. Error bar: standard deviation (SD). Significant difference (*).

Figure 4 shows the IFN-γ index values obtained at 9 mpv from Group B after stimulation of the blood with different antigens. These were significantly higher among the vaccinated subgroups stimulated with avian PPD, bovine PPD, or johnin (*p* < 0.05). However, when the blood was stimulated with antigens derived from *M. bovis*, significant differences (*p* < 0.05) in the IFN-γ index were observed within the subgroups in which the blood was incubated with bovine PPD, with no differences between the three age subgroups for the values obtained with the PC antigens (Figure 4).

**Figure 5:**
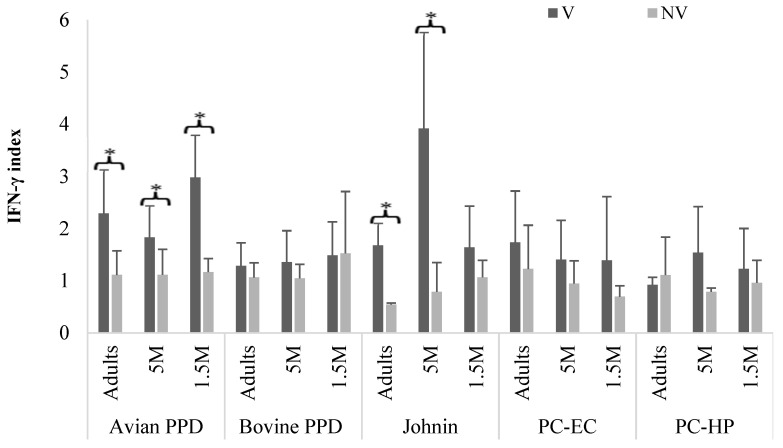
Group B. Mean and SD values of the IFN-γ index in animals of the different age subgroups, according to the antigen used to stimulate the blood, at 15 mpv. M: months old; mpv: months post vaccination; V: vaccinated; NV: not vaccinated. Error bar: standard deviation (SD). Significant difference (*).

In the sampling carried out at 15 mpv (Figure 5), in the animals whose blood was stimulated with avian PPD or with johnin, the differences in the mean values of the IFN-γ index remained significant (*p* < 0.05) between the vaccinated and control subgroups in the Adult V and 5 MV groups. However, in the 1.5 MV subgroup, significant differences with the controls were only observed when the blood was stimulated with avian PPD. Between both antigens, differences were seen only in the 5 MV subgroup, where the IFN-γ levels were significantly higher after johnin stimulation (*p* < 0.05) (Figure 5). 

When the blood was stimulated with *M. bovis* antigens, no differences were found in the IFN-γ production between the vaccinated animals and controls in any of the age subgroups and for all three antigens tested, including bovine PPD.

## 4. Discussion

One of the main limitations of the use of vaccination as a paratuberculosis control method are the cross reactions that occur in the tuberculosis diagnostic tests used in the eradication campaigns [5,7,20], which have led to the prohibition of vaccination in bovine species in many countries, including Spain, although is permitted in goats [3,4]. 

In this work, goats were used as an experimental model for various reasons: they offer similarities to cattle, such as the high sensitivity to tuberculosis infection suffered by both species [21,22], they are subject to eradication campaigns covering different age ranges in a flock, and the existence of cross-reactions has also been demonstrated among goats vaccinated against paratuberculosis in field and experimental studies [3,6].

Paratuberculosis infection and vaccination trigger a cellular immune response measured using the IGRA test [19,23,24,25,26,27,28,29,30,31], in which PPDs are employed, as with IDTB, where cross-reactions can appear in cases of tuberculosis infection [3,6,32,33].

The causative agent of paratuberculosis, encompassed in the *Mycobacterium avium* (MAC) complex [34], has a high phylogenetic homology with *M. bovis* and could enable cross-reactions when bovine PPD is used for tuberculosis diagnosis [35]. 

For the diagnosis of tuberculosis, IDTB is the standard test, which can be simple if only bovine PPD is used, or comparative if avian and bovine PPD are used [4]. The IGRA is a complementary test to the IDTB, which is also employed in the tuberculosis eradication campaigns [4,36], and was chosen in this study to assess different mycobacterial antigens due to the ease of access to the blood samples, less handling of animals, and the fact that IDTB in goats, under field conditions, is subject to official control.

In both IDTB and IGRA tests, the existence of nonspecific reactions in animals vaccinated for paratuberculosis has previously been reported in the diagnosis of tuberculosis in cattle and goats [3,6,7]. The results of the IGRA in this study have confirmed these cross-reactions when using PPDs, since in all of the animals vaccinated against paratuberculosis, there was a response against them, including bovine PPD. However, this response was less intense than that found using avian PPD or johnin, as other authors previously reported in cattle and goats in field conditions [3,8,37]. However, there are some studies in bovine species where a similar response for avian PPD and bovine PPD was found [13,32].

In any case, in our study, as in others, vaccination induced a response against bovine PPD of variable duration. This response had already disappeared in the 9 and 15 mpv samples in Groups A and B, respectively, in agreement with other field studies using IDTB [3]. However, in other previous works carried out in goats, this response persisted up to 23 mpv [37] or even up to 3.5 years [38]. In cattle, the differences would have disappeared at 24 mpv [32]. Therefore, these interferences would have a limited duration in time, a fact to take into account when simple IDTB is practiced in herds vaccinated with paratuberculosis. In any case, this study confirms the existence of a nonspecific response in the IGRA test if only bovine PPD is used for the diagnosis of tuberculosis.

In recent years, some work has been carried out aiming to search for new and more specific antigens that would allow the differentiation of animals vaccinated against paratuberculosis from animals infected with tuberculosis [6,12,13,14,15,16,17,18,39,40]. Among them, the new specific antigens of *M. bovis* ESAT-6, CFP-10, and recombinant proteins (Rv3615c, RV peptide pool) stand out, which are part of the peptide cocktails (PC-EC and PC-HP) used in this study. Recently, a new recombinant proteins pool derived from ESAT-6 CFP-10, PPE26, PPE60, and the PiRG family has shown good results in a reduced number of calves vaccinated against paratuberculosis, but they have not been compared to johnin and are scarcely tested in goats in vaccinated and infected animals [13].

In a recent study in calves and goats [14,15], peptides of tuberculosis ESAT6, CFP10, and RV3615c have been shown to be more specific than bovine PPD and johnin in experimental infected, but unvaccinated, animals against Map. Identical results to ours were obtained in a vaccination experiment against paratuberculosis in goats that were later infected with *M. caprae* [6], where it was found that, at 3.5–4 mpv, the stimulation of peripheral blood with these antigens derived from *M. bovis* did not give any type of reaction, while when the infection by *M. caprae* occurred, there was a specific reaction against them. According to these results, in an infection with *M. bovis* in calves that had previously been vaccinated against paratuberculosis, but not infected with Map [8], non-specific responses were obtained in those vaccinated animals when the samples were stimulated with the same antigens used in our study. As already indicated, its specificity in the diagnosis of tuberculosis in animals infected with *M. bovis* or *M. caprae* has already been verified in different studies using both the IGRA test and the IDTB [6,8,14,15,16,17,18,41,42,43].

Another objective of this work was to evaluate the response obtained in the IGRA test when stimulating the blood of animals vaccinated or experimentally infected with paratuberculosis with two proteins purified from Map (VK 055 and VK 067) and to compare it with that of the two PPDs, the avian, and the johnin. However, when compared with PPDs, significant differences were only obtained with johnin, not with avian PPD. It seems that this protein does not allow an earlier detection of Map infection than with PPDs, at least under the conditions of this study where IFN-ɣ production was only assessed at 3 months post infection (4 mpv), since no differences were found in the responses in the group of infected and unvaccinated animals from Group A.

Different Map proteins, such as Map_0268c and Map_3651c, have been shown to be capable of inducing a cellular immune response measured with the IGRA technique in cattle experimentally infected with Map [14], goats [15], or in sheep subclinically infected in natural conditions, although of a lower intensity than the response achieved against johnin [16]. Latency proteins have also been used (LATP-1, LATP-2, and LATP-3) that have given optimal results, also in IGRA, but always below the productions compared to johnin [44]. The same result has been obtained with a Map membrane protein (L5P) in cattle [45] that stimulated IFN-γ production in infected animals, although below avian PPD, too. At present, as with the proteins tested in this study, neither seems to be more specific or sensitive than both PPDs for the identification of Map-infected animals.

Regarding avian PPD and johnin, a greater production of IFN-ɣ was detected when stimulating the blood in vaccinated animals with both antigens, as previously reported [15,25,26,30,33,46], with no significant differences between them, or even better results when using avian PPD compared to the johnin in calves infected by Map [46], which would support its indistinct use. In the animals from Group A, these differences between the vaccinated and control animals had already disappeared at 9 mpv, while those from Group B still persisted at 15 mpv. It is true that the ages of the animals varied between both groups, and it can be thought that the vaccination of young animals (1.5 months) would make the response less durable; in the goats from Group B that were vaccinated at 1.5 months, IFN-ɣ production was still higher at 15 mpv, but only with avian PPD. In another study [35] where young 1-month-old kids were also vaccinated, the cellular immune response to vaccination was maintained up to 23 mpv. The length of the protein chain that is part of these new proteins could be an important sensitivity factor and therefore play a role in discriminating those vaccinated over time [13].

As has just been pointed out, in Group B, it was found that, in the animals vaccinated at 1.5 months, avian PPD continued to induce a specific response, but johnin did not. This result could be related to the fact that the cellular response in animals vaccinated at a younger age (1.5 months) would be less durable than if they are vaccinated later [25], and that johnin could have a lower stimulus capacity than avian PPD, despite being more specific when goats are vaccinated at different ages. 

## 5. Conclusions

The use of any of *M. bovis* antigens (PC-EC and PC-HP), instead of bovine PPD, looking for discrimination between false reactants in tuberculosis diagnostic tests and vaccinated animals against paratuberculosis in different age ranges, or possibly because they are infected by Map, may prove promising in the future if their use in diagnostic tests for tuberculosis can be standardized and incorporated into official techniques. Further studies, including the IDTB test, and also more animals, would be necessary to validate their use as substitutes for bovine PPD. Nevertheless, at the moment, its high commercial costs would make its use in large-scale campaigns unfeasible. In contrast, the Map antigens tested do not seem to provide any advantage over PPDs under the conditions of this work.

## Data Availability

Not applicable.
